# Mechanical, Electrical, and Thermal Characterization of Pure Copper Parts Manufactured via Material Extrusion Additive Manufacturing

**DOI:** 10.3390/ma15134644

**Published:** 2022-07-01

**Authors:** Antonio Cañadilla, Ana Romero, Gloria P. Rodríguez, Miguel Á. Caminero, Óscar J. Dura

**Affiliations:** 1 E.T.S. Ingeniería Industrial (ETSII), Instituto de Investigaciones Energéticas y Aplicaciones Industriales (INEI), Universidad de Castilla-La Mancha (UCLM), 13071 Ciudad Real, Spain; antonio.canadilla@uclm.es (A.C.); gloria.rodriguez@uclm.es (G.P.R.); 2 Escuela de Ingeniería Industrial y Aeroespacial (EIIA), Instituto de Investigación Aplicada a la Industria Aeronáutica (INAIA), Universidad de Castilla-La Mancha (UCLM), 45071 Toledo, Spain; ana.rgutierrez@uclm.es; 3 E.T.S. Ingeniería Industrial (ETSII), Instituto Regional de Investigación Científica Aplicada (IRICA), Universidad de Castilla-La Mancha (UCLM), 13071 Ciudad Real, Spain; oscar.juan@uclm.es

**Keywords:** material extrusion additive manufacturing, copper, metal powder filament, sintering, mechanical characterization, electrical and thermal characterization

## Abstract

Material Extrusion Additive Manufacturing (MEAM) is a novel technology to produce polymeric, metallic, and ceramic complex components. Filaments composed of a high-volume content of metal powder and a suitable binder system are needed to obtain metallic parts. Thermal and energetic controversies do not affect MEAM technology, although common in other additive manufacturing (AM) techniques. High thermal conductivity and reflectivity of copper to high-energy beams are the most challenging properties. A material extrusion technique to produce high density and quality copper parts is deeply studied in this research. Characterization of the filament, printed parts, brown parts and final sintered parts is provided. The sintering stage is evaluated through density analysis of the sintered copper parts, as well as their dimensional accuracy after part shrinkage inherent to the sintering process. The mechanical behavior of sintered parts is assessed through tensile, hardness and impact toughness tests. In addition, the measured electrical and thermal conductivities are compared to those obtained by other AM technologies. High-density components, with 95% of relative density, were successfully manufactured using MEAM technology. Similar or even superior mechanical, thermal and electrical properties than those achieved by other 3D printing processes such as Electron Beam Melting, Selective Laser Melting and Binder Jetting were obtained.

## 1. Introduction

Copper, thanks to its specific properties, is widely used as raw material for several industry and engineering sectors such as aerospace, power generation, defense, electronics, among others. It is predominantly used for electrical conduction services and thermal management due to its excellent electrical and thermal conductivity, and its moderate price compared to gold and silver [1,2,3,4,5]. Moreover, copper parts exhibit a good corrosion resistance, good machinability, and antibacterial performance, so these properties make copper a suitable material for automotive and construction applications, or even medical devices. It is used in the fabrication of numerous components such as heat exchangers, electronic connectors, plastic deformation tools, springs and bearings, propulsion devices in aeronautic features, cooling channels and gas turbines. Nowadays, the fast pace of industrial growth requires more complex geometries, structures, and optimal properties of copper components [6]. 

Over the last decade, the development of additive manufacturing (AM) technologies has attracted interest in both the academic and industrial sectors thanks to its ability to produce metal parts with challenging geometries, without the need to use post-processing operations and minimizing waste products [7,8,9]. Contrary to conventional subtractive manufacturing methodologies, additive manufacturing is based on a bottom-up process, in which metal parts with arbitrary geometry are created layer by layer [10,11]. Additive manufacturing of metals allows high customizability, and it is competitive to other conventional manufacturing methods such as machining, metal casting or welding [12,13], so they are becoming more and more important in several industrial sectors. Complex components like objects with internal structures, thin walls, or porous parts can be additively fabricated without considering design restrictions due to machining tool limitations. Moreover, the geometry optimization via 3D software offers many benefits like complicated geometries, shorter manufacturing times, multifunctional integration, and savings in structures’ weight [14]. In addition, the option of doing post-processing methods, for example hot isostatic pressing (HIP) [15,16] or abrasive polishing [17], can further enhance mechanical and functional properties of printed metal parts.

Over recent years there has been a huge development of the additive manufacturing processes of metal feedstocks, including copper. Direct energy deposition processes and powder bed fusion systems are the methodologies most employed for the fabrication of complex metal parts in short production series. The most studied processes are electron beam melting (EBM) and selective laser melting (SLM), which have been applied in the manufacturing of components of steel, nickel, aluminium, and titanium alloys, among others. In the case of pure copper parts, the 3D printing of components with high thermal and electrical properties has been extensively studied for their implementation in thermal management systems [18,19], electronic devices [20,21,22], and in the aerospace industry [23,24]. However, the manufacturing of complex shape parts with low porosity using laser-based AM techniques is challenging due to the thermal properties of copper and copper alloys [25]. The area melted by the high-energy beam experiences quick heat dissipation due to copper’s high thermal conductivity, which harms the manufacturing process. Thermal issues such as layer curling, high local thermal gradients, part deformations, delamination, and failure are consequently more probable due to thermally induced stresses [25]. Moreover, low deposition of energy in the material is experienced because of the reflectivity of copper to laser light, resulting in SLM copper parts with low density and poor surface quality. Furthermore, this phenomenon is harmful for the laser and its optics [26,27]. The use of electron beam melting (EBM) techniques for the manufacturing of copper and copper alloys pieces solves the previous limitations thanks to the absence of optical reflectivity of the materials to the electron beam [28]. The energy deposited is enough to consolidate copper parts, but the high thermal energy dissipation during the melting stage could cause defects such as porosity, cracks, internal stresses, or shape distortion [29]. Heating the powder bed could reduce those defects. However, it has to be mentioned that high-cost infrastructure is required to handle explosive powders safely and the beam-based AM equipment. Thus, SLM and EBM require techniques that are expensive and complex to operate [30,31].

These drawbacks underscore the need for more economical alternative metal 3D printing techniques, with lower production costs and energy consumption. Copper parts can be produced by several solid-state metal additive manufacturing technologies [32], which are classified based on the bonding mechanism and its effect on the microstructure and mechanical properties of the resulting components. Although many solid-state AM technologies are currently being explored, only some of them have shown good results in copper processing. These include three techniques based on mechanical strain and two on sintering: additive friction stir deposition (AFSD) [33], ultrasonic additive manufacturing (UAM) [34], cold spray [35], binder jetting (BJ) [36], and material extrusion additive manufacturing (MEAM) [37], respectively. The mechanical strain-based techniques use mechanical energy to facilitate metallic bonding. Therefore, the mechanical disruption of the oxide layer is followed by material bonding through severe plastic strain, which can be induced by friction, ultrasonic scrubbing, or supersonic impact, of powder particles onto a substrate or a previous layer of the same material. On the other hand, sintering-based solid-state additive manufacturing techniques consist of the consolidation of a 3D-printed powder compact, heating at a temperature below but close to the melting temperature. The externally applied thermal energy increases the total system energy, causing the reduction of the total material surface area and the metal particle bonding via the atomic diffusion occurring between them. In binder jetting, one or more inkjet printheads deposit a liquid binder agent on the top of a powder bed to selectively join the particles forming a printed cross-sectional layer. This operation is repeated until the final part, called “green part”, is completed [38]. Then, the printed part is subjected to a thermal cycle where the binder system is removed, and the powder particles are strongly bonded by sintering process in order to achieve final density and strength of the material. The sintered components achieve an approximate relative density of 85%, so post-processing is needed to achieve full density. In addition, BJ equipment is more expensive than MEAM equipment [38], so this last manufacturing technique could be the most economical alternative for low and medium production volume of complex medium-sized copper parts. 

The Material Extrusion Additive Manufacturing technique is based on an AM process well developed and used globally to produce 3D parts from filaments extruded through a small diameter nozzle onto a heated platform, called Fused Deposition Modeling (FDM) [39,40]. This technology can be used to manufacture complex parts of thermoplastics [41,42], fiber filled composites [43], low melting point metal alloys [44], even copper [45], high-melting-point metallic alloys [46,47,48], cermets [49,50] or ceramics [51,52]. Instead of melting the powder particles, as in SLM and EBM, these are combined with a binder system in a filament, which is selectively extruded and deposited following the CAD model with the design of the target part. The first layer of material is extruded onto a platform and then the process is repeated layer by layer until the final geometry is obtained. Next, the product is subjected to a solvent and/or thermal treatment to remove the binder system. Finally, a sintering cycle is performed, and powder particles are bonded. The MEAM parts are usually placed in a conventional electrical furnace to remove the last binder components, coalesce the metallic particles thank to diffusion bonding, and densify the shaped specimen [44,46,48]

Several studies have demonstrated that MEAM technology can be employed to produce a large variety of metal alloys and ceramic materials using binder and powder compositions. MEAM technology to manufacture metal and ceramic components is also known as a printing-debinding-sintering (PDS) process, fused layer modelling (FLM), or fused filament fabrication (FFF) [53,54]. MEAM is becoming one of the most used 3D printing techniques and to process a huge variety of highly filled materials. This method requires a much lower initial investment than SLM or EBM technologies, and has short processing times, an easy operating system, control on processing parameters, low material wastage, and the chance to simultaneously use numerous materials. The MEAM process does not need an enclosed chamber. It is a scalable technology, and no expensive energy sources are required, and, moreover, it is not affected by the melting point, optical reflectivity, reactivity and/or thermal conductivity of the processed material. In some metal MEAM developments, 17-4PH steel, H13 tool steel, or 316L stainless steel parts were obtained via commercially available 3D printers [55,56]. Although extrusion 3D printing and sintering of pure copper have been explored in several studies, the research works on microstructure, porosity, geometric, physical, and mechanical properties of the final copper parts obtained via MEAM are scarce. 

Singh et al. [57] manufactured 3D-printed copper parts by extruding feedstock with a screw extruder similar to that used in MIM process. A maximum green density of 5.5 g/cm^3^ and 1.6 µm of minimum surface roughness were achieved modifying the printing parameters (nozzle speed, extrusion temperature, layer thickness, etc.). A solvent debinding by immersion of the samples in water and a subsequent thermal debinding at 500 °C for 1 h were carried out. The parts were sintered in a cycle in two stages: the first at 950 °C for 3 h, and the second at 1030 °C for 3 h, as suggested by another study [58]. Final parts with a relative density of ≈88%, without significant printing defects, except for the porosity inherent to the process, were obtained. However, this density value is lower than the value obtained by the conventional MIM process, so further optimization is required. Hong et al. [59] and Yan et al. [60] analyzed the manufacture of copper parts with a raw material composed of a copper polyvinyl carboxy polymer and polyvinyl alcohol paste-filament. The effects of printing parameters were investigated to achieve acceptable green parts, in which copper content, retraction distance, layer height and extrusion volume are included, among others. After sintering at 1050 °C, a shrinkage of 45% took place and a relative density of 87% was registered in the copper products. Zhao et al. [61] have also proved the viability of extrusion technology to make copper parts, but the microstructure and final properties of the parts were not reported. Ren et al. [62] suggested a sintering stage close to 1000 °C to obtain final parts with higher relative density, near to 90%, but a low binder fraction remained in the final products. Some advantages of using filament feedstocks are low-cost equipment for the printing stage, availability of powders used in powder metallurgy, and the same feedstocks used in metal injection molding (MIM) or metal extrusion additive manufacturing (MEAM) [63,64,65,66]. For the use of filaments as feedstock, their binder systems must have an adequate flexibility and mechanical strength so they could be used in commercial MEAM printers. This way, new binders are being developed, in which copper and other metal powders can be found [67,68,69]. 

In accordance with the current state of the art, this research is based on the processing of copper MEAM components, seeking to obtain high-density and quality parts, minimizing defects, as well as to provide a detailed geometric, mechanical, and physical performance study. The achieved results are compared to those obtained by other copper manufacturing processes, such as SLM, EBM, Binder Jetting and wrought copper, to validate the MEAM scope and actual viability of the technique. The current study provides a general view and relevant information regarding the mechanical and functional behavior of pure copper components manufactured via the MEAM technology, which may be helpful for expanding the applicability of this technique in engineering solutions that require the use of copper.

## 2. Materials and Methods

### 2.1. Material, MEAM Processing and Suitable Specimen Selection

**Figure 1:**
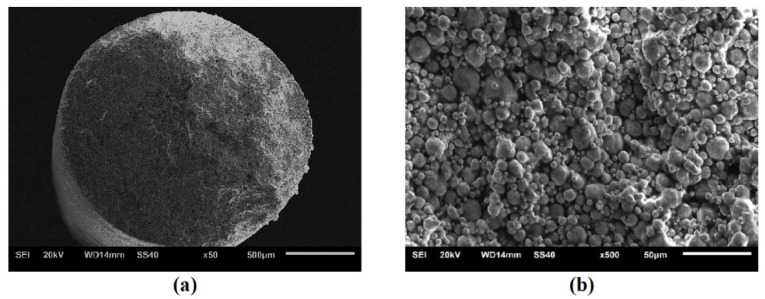
SEM micrographs of the copper filament cross section. (**a**) Details of the inner configuration of filament showing its cylindrical geometry. (**b**) Details of copper powder morphology and the binder system.

A pure copper powder and polymer filament, with a metal fraction of over 95 wt.% and a 1.85 mm in-diameter, supplied by Markforged with designation F-MF-1010, was used [69]. The MEAM technology is divided in three main stages: printing, debinding and sintering. Figure 1 shows the SEM micrographs of the copper filament, in which can be appreciated the spherical metal powder particles, with irregular sizes in the 10–20 µm range, coated by the binder-system that adheres particles with each other. The filament is not covered by any polymeric external coating.

**Figure 2:**
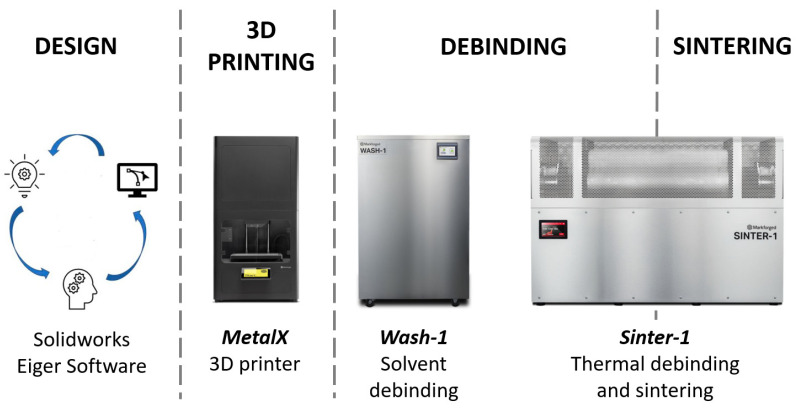
Flow chart of the manufacturing process of copper parts with Metal Extrusion Additive Manufacturing technique using the Markforged equipment.

Pure copper parts were fabricated using the Markforged equipment, that englobes a 3D metal printer (MetalX), a washing machine which works with dissolvent (Wash1) and a tubular furnace with a tight temperature control between room temperature and 1300 °C (Sinter1). Figure 2 shows the equipment that have been used and the manufacturing process which has been followed in this work. 

**Figure 3:**
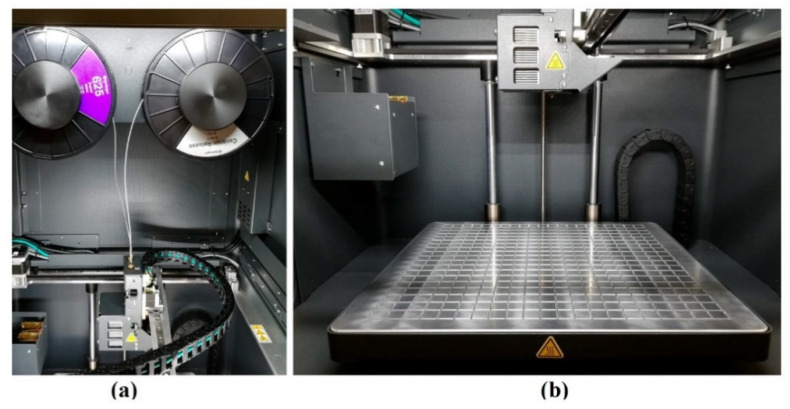
MetalX 3D printer. (**a**) Material storage chamber and (**b**) printing chamber.

The first stage consists of the 3D printing of copper specimens using the MetalX 3D printer, which allows a manufacturing volume of 300 × 467 × 1120 mm^3^, and a layer thickness between 50 and 125 µm. Figure 3 shows in detail the 3D printer that has been used in this study. This equipment works with two different filaments: the metal filament of interest, in this case of copper, and a ceramic filament, which is used to build the auxiliary supports if these are needed during the printing process. The metal and ceramic filaments are carefully located in their specific coil at the upper printer cavity (Figure 3a), which is thermally controlled in the suitable conditions for the raw materials. The print head with double extruder (metal and ceramic) and the print bed are situated inside the printing chamber (Figure 3b), which is also heated during the manufacturing process. The solvent debinding process is carried out in the washing equipment, which has a work volume of 356 × 254 × 203 mm^3^. For the final thermal debinding and sintering stages, a tubular furnace is used, which can heat up at a maximum temperature of 1300 °C and has a controlled atmosphere reaction chamber, whose volume is 18,356 cm^3^ (145 mm in diameter and 1112 mm in length).

Copper parts with different geometries were manufactured to analyze their geometric and mechanical properties, as well as their electrical and thermal conductivity. Cylindrical and prismatic specimens were processed to analyze the relative density and the volumetric shrinkage after the printing and sintering processes, respectively. In addition, tensile and impact specimens were elaborated according to ISO 2740 [70] and EN ISO 5754 [71] standards, respectively. Moreover, cylindrical copper parts with 15 mm diameter and different thicknesses were used for the determination of thermal conductivity of the MEAM parts, while square-based copper parts were used to evaluate their electrical conductivity.

### 2.2. Processing Parameters and Printing Strategy

**Figure 4:**
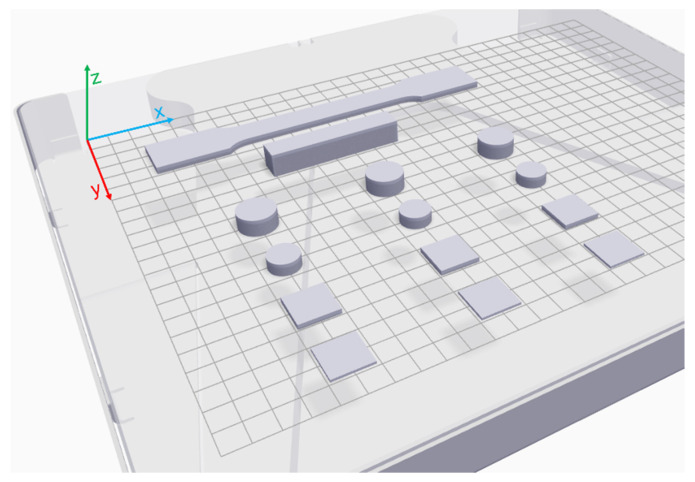
Printing strategy followed in each sample.

The manufacturing process starts with the CAD design of the geometry of the copper part to be printed, using computer assisted design software (Solidworks, in this case). When the CAD model is done, Eiger software is used to divide the model part in the specific layers to be printed on. Another task that the software performs is the oversizing of the part considering the contraction that it will suffer during the sintering process, which depends on the material and the geometry. Eiger software also allows the setting of some parameters for the impression, such as the printing scale, filling type (solid or triangular structure), layer thickness, exterior wall thickness, and the use of rafts to ensure adherence and enhance stability during the 3D printing. The software is useful for setting up the parts orientation on the working platform for the printing; the user should consider the geometry avoiding, as far as possible, extra supports and economizing on the manufacturing process. In general terms, the larger dimension is placed on the *X* axis, the shorter one on the *Y* axis and the different layer are printed following the *Z* axis. The printing strategy followed in each sample is shown in Figure 4. When all the parameters and considerations are selected, it is possible to check the total printing time, the duration of the solvent washing cycle, the approximate cost of the used material, and the printed and sintered parts dimensions. The parameters established for the copper 3D printed parts manufacturing are shown in Table 1.

The geometric and printing data are transferred to the 3D printer that, following the designed printing process, prints the metal in the position and with the specifications specified in the layered model, starting on the printing bed, and growing up the geometry layer by layer. Each layer is printed following two deposition strategies. Firstly, the filament is extruded following the perimeter of the layer using a contouring strategy and, secondly, the infill of the part is done with a zig-zag solid infill strategy that is deposited perpendicularly respect to the previous layer. The contouring thickness is defined when the external wall parameter is established in the previous design step. The print head does the horizontal movements in *X* and *Y* axis, while the print bed does the vertical displacement in the *Z* axis, so the designed geometry can be printed using the selected metallic material. Both metal and ceramic extruders work with an extrusion temperature of approximately 220 °C, which is adequate for avoiding filament obstructions or excessive fluidity.

The printed components are called green parts and contain both metal powder and binder. Next, two debinding stages are carried out to remove the polymeric component. The first consists of a chemical removal of the paraffin wax by the immersion of the material in OpteonSF-79 solvent using the washing equipment. Green parts are submitted to a solvent debinding iterative cycle with static conditions to remove approximately a 2.8 wt.%, that is the paraffin wax mass proportion in the printed material. After each cycle, the parts are dried in ambient conditions for 4 h and weighed. The process is repeated until the measured mass at the end of each iteration is stable, since this is an indication that the solvent debinding of the paraffin wax is completed. Eiger software provides an approximate time for the solvent debinding stage, based on the mass and geometry of the parts, but the effectiveness of the process must be corroborated experimentally. After this process, brown parts are obtained, which are brittle and must be manipulated carefully.

The second debinding process consists of a thermal treatment for the removal of the high molecular weight (HMW) polymer that remains in the brown parts. The thermal debinding is included in a cycle that also encompasses the sintering process and the controlled cooling, necessary for the obtaining of the final metal parts. The thermal cycle is divided into 3 main stages: a first heating to the HMW polymer debinding temperature, which is maintained for a certain time to ensure the total binder system removal; a second warm up to the copper sintering temperature with its correspondent plateau; and, finally, a controlled cooling until room temperature. The complete thermal cycle is 30 h long and is carried out under protective atmosphere conditions: 2.8% H_2_–97.8% Ar reducing mixture atmosphere is used in the thermal debinding stage, and a pure Argon atmosphere in the sintering stage and the controlled cooling. The final pure copper metallic parts are obtained after the complete thermal cycle.

### 2.3. Experimental Set-Up

The microstructural quality of the final copper parts at the different processing stages was assessed using a LEICA DM IRM Optical Microscope and a JEOL J8M6500 Scanning Electron Microscope. There are several methods to determine density values of the parts, but in this study, Archimedes method has been selected because it is easy to apply and has high reproducibility [72]. Relative density is measured by Archimedes method according to ISO 2738 [73], using the mass of the sample in air and submerged in water. Green and sintered parts with prismatic geometry are measured by the 3-coordinate measuring device per contact Etalon Derby Tesa to evaluate the shrinkage and dimensional tolerances. Mechanical properties of copper parts were analyzed through Vickers microhardness, tensile and impact energy-absorption tests. The Vickers microhardness was measured using a testing load of 50 g and a dwell time of 10 s. Ten microhardness measurements were taken in different positions of the polished cross-section of each sample to calculate the mean value. The tensile behavior, including ultimate tensile strength, yield strength and effective elastic modulus, is evaluated with Microtest multiaxial equipment, using a 50 kN load cell in quasi-static conditions at a rate of 25 N/s, according to ISO 3325 [70]. The impact toughness is analyzed by HOYTOM Charpy equipment according to EN ISO 5754 [71], using an impact energy of 300 J. Both tensile and impact energy tests were repeated three times and the mean results were calculated. The fracture surfaces after the tensile and Charpy tests were examined utilizing SEM to analyze the type and mechanism of fracture of Cu MEAM parts.

For the determination of the electrical conductivity, the Van Der Pauw Method was applied, which employs a four-point probe placed around the perimeter of the sample. The Van Der Pauw technique accurately measures the electrical resistivity of an arbitrary-geometry part and facilitates its resistivity, which becomes important for irregular shapes [74]. Square-based copper parts with different thicknesses were manufactured to meet the condition of the Van Der Pauw method: the samples are uniformly thick, the surface of each sample is singly connected (without isolated holes), and the four contacts are at the perimeter of the samples and are sufficiently small [75]. The electrical resistivity ρ [Ω·m] is obtained by Equation (1), where *R* [Ω] is the average of the measured electrical resistance by alternating source-measure contacts, *t* [m] is the thickness of the specimen and f is the form factor associated with the sample cross-section (≈1 for square cross section). Electrical conductivity σ [S/m] is obtained as the inverse of electrical resistivity. The calculated electrical conductivity is compared with respect to IACS (International Annealed Copper Standard), where the reference value of 100% IACS of the annealed pure copper is 58.106 S/m [76]).

(1) $$\rho =\frac{\pi \cdot t\cdot f}{\ln\left(2\right)}\cdot R$$

The thermal conductivity is determined through laser flash analysis with LFA-1000 system from Linseis Company. To make sure the reflectivity of the printed copper would not affect any of the incoming laser flash, the test sample is coated with dry graphite film as a “flat black”. A series of three laser flashes is shot into the cylindrical copper parts, with first low, then medium, and finally full power of the laser. The average of these three tests gives the thermal diffusivity measure. The thermal conductivity can be calculated using Equation (2) [77,78]. *λ* [W/mK] represents the thermal conductivity, *α* [cm^2^/s] is the thermal diffusivity which is the obtained value from the laser flash device, $\rho_{rel}$ [g/cm^3^] is the material density and *C_copper_* [J/gK] is the specific heat. The specific heat is assumed constant at 0.388 J/gK for pure copper. Therefore, the sample density has the highest influence on the thermal conductivity, which is measured by the Archimedes method according to the standard.

(2) $$\lambda =\alpha \cdot \rho_{rel}\cdot C_{copper}$$

## 3. Results and Discussion

### 3.1. Copper Parts Manufacturing

**Figure 5:**
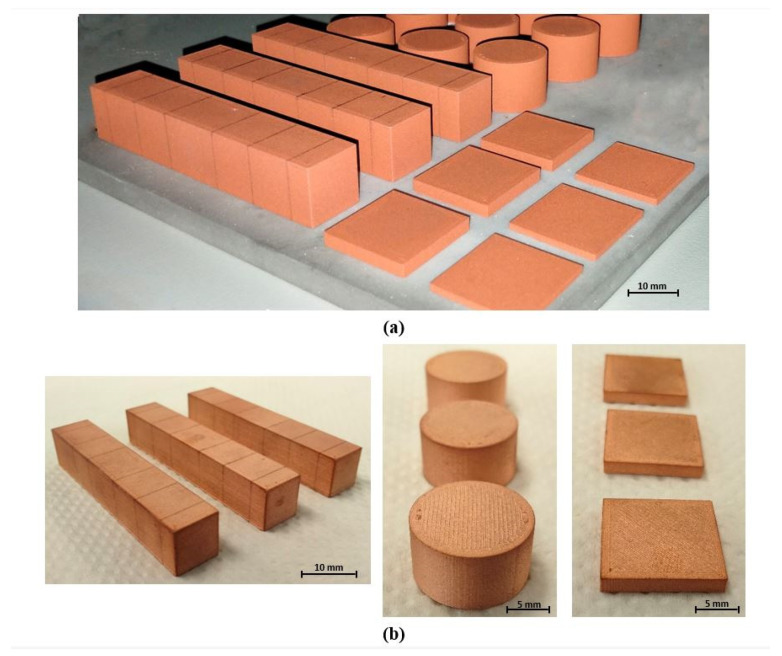
Copper parts manufactured by Metal Extrusion Additive Manufacturing. (**a**) Green parts after printing stage. (**b**) Final sintered parts.

Pure copper parts with different geometries were manufactured following the MEAM technique, and some of them are shown in Figure 5 in different stages. Regarding the printing stage, all the designed parts could be printed successfully without apparent geometry distortions or defects, and the employed time and material depended on the established printing parameters and part shape. The metal filament is a combination of pure copper powder and binder, which allows the manufacturing of the desired geometry and assures the stability of the green part until the next process stage. Most commercially available highly-filled polymers with metal powder are used in Metal Injection Molding (MIM), and they are designed to have a good flowability so the cavity of the injection molding tool can be completely filled [79]. Zhong et al. [80] reported that the main properties of the filament material for MEAM technology must be enough strength and stiffness, good ductility, and flexibility, so the filament can be wound in a spool for the continuous feeding and can be well-processed by the printing machine. The filaments used have a binder system made up with wax, which is chemically removed after the printing process, and a high molecular weight (HMW) polymer, which is thermally removed in the pre-sintering process. This binder system is homogeneously combined with pure copper powder to form the filament material, which has approximately the following proportions: 2.85 wt.% of wax, 2.15 wt.% of HMW polymer, and 95 wt.% of metal powder.

Figure 5a shows several green specimens with different geometries, just after the print process. These had a non-metallic appearance due to their binder content but exhibited an enough strength to be easily manipulated. After the debinding stage, through the solvent washing cycle, all the specimens reached a weight loss of 2.8 wt.%, so the wax included in the binder system was successfully removed. The washed parts, called brown parts, had similar color and consistency to the green parts, and these could be easily handled to continue the manufacturing process. Finally, the thermal debinding and sintering cycle of the parts was for 30 h. As can be observed in Figure 5b, the sintered copper parts achieved the characteristic metallic aspect, the geometry of each specimen remained stable, and no evidence of oxidation was detected. All the manufactured copper parts exhibited strong and high-quality metallic appearance, with no signs of melt areas or lack of sintering. Moreover, the final metallic specimens show in their surface the characteristics of the 3D printing process with fine printing lines. It can be appreciated how the print head deposited the material following the layer perimeter and then the inside part was filled with parallel lines. This manufacturing strategy is similar in the printing of all parts independently of their shape or geometry.

In addition to the change in appearance, the copper specimens experienced a volumetric shrinkage after sintering, which influences in the dimensional tolerances of this manufacturing process. The dimensions of the parts obtained via the 3-coordinate measuring device are used as the basis for calculation of the volumetric shrinkage and the dimensional tolerances of the pure copper parts after the thermal process, as shown in Table 2. The measurements have been taken in each printing axis (X, Y, Z), so the linear shrinkage percentages after the sintering process have also been calculated independently for the three axes. As can be observed in the presented results, the shrinkage that copper parts suffered is slightly greater in the Z-direction than the X or Y directions. The X and Y dimensional shrinkages are very similar, namely, 13.2% in the *X*-axis and 13.4% in the *Y*-axis. The larger shrinkage in the *Z*-axis of 13.8% may be attributed to gravity effects that become more important during the sintering process. This variation proves that it is important with this technique to consider the dimensional compensations needed in the sizing of the part in each direction. 

The dimensional tolerance has been calculated using the nominal dimensions established in the CAD model and the real measurements of the sintered copper parts, applying Equation (3). When the obtained tolerance values are positive, it means that the measured value of the sintered part is above the nominal dimension defined in the CAD model. On the other hand, negative values imply the copper part had contracted more than initially established in that direction.

(3) $$∆D\left(\%\right)=\frac{Di_{sintered}-Di_{CAD}\;}{Di_{CAD}}\;$$

The tolerance values presented in Table 2 allows the analysis of the XY-plane, where the *Y*-axis showed fitter results than the *X*-axis, at 0.024% and −0.151%, respectively. Taking into account that the Y-dimension is considerably shorter than the X-dimension in the evaluated copper part, this behavior could be based on the manufacturing error introduced by the print head. When the displacement of the print head for the material deposition is large, it causes a higher error accumulation, which leads to wider dimensional tolerances after the sintering shrinkage of the metal parts. Attending to the tolerance percentage obtained for the Z-measurement (0.08%), this is slightly larger as compared to the X-direction, even both dimensions are nominally equal. These dimensional differences in sintered parts have been previously reported and are partially attributed to the different degrees of particle consolidation across layers and within layers [17]. The printing process in the MEAM method starts with the deposition of the first layer in the XY-plane, and then the part is built up layer by layer in the Z-direction. This printing strategy means that the layer-by-layer interaction, the filament solidification after its deposition, and the sintering process itself, influence the nominal deviations in the *Z*-axis, which therefore decreases its precision. 

**Figure 6:**
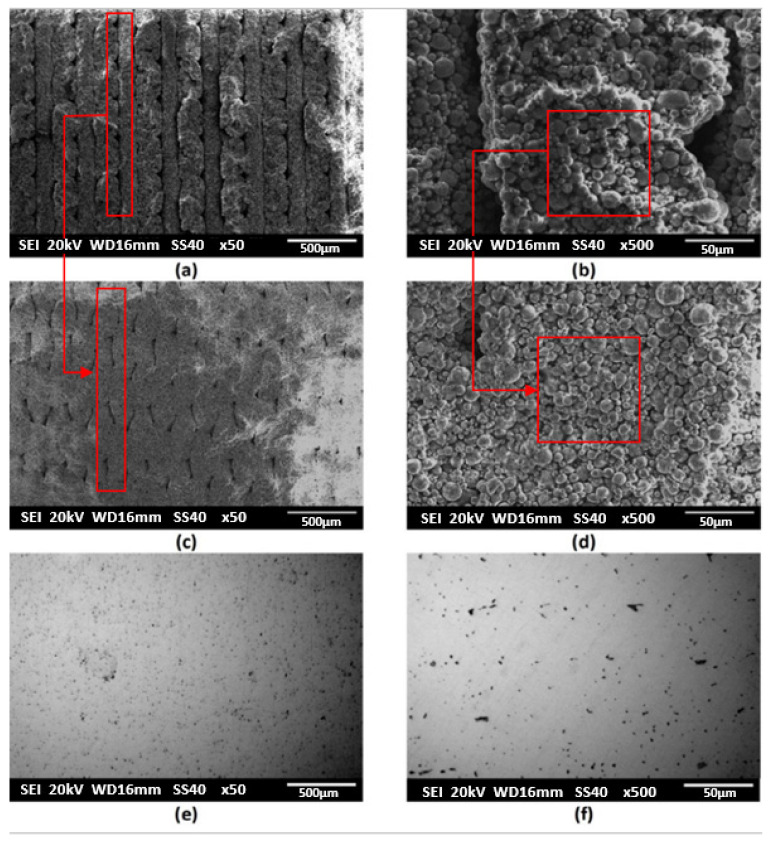
SEM images of (**a**,**b**) green part, (**c**,**d**) brown part and (**e**,**f**) sintered final part. (**a**,**c**,**e**) ×50, (**b**,**d**,**f**) ×500. (**a**–**c**) Detail of the porosity inherent of the layer-to-layer manufacturing process. (**b**–**d**) Detail of the porosity inherent of the filament due to the distance between metal particles.

The structure of the samples in all MEAM process manufacturing stages have been microscopically evaluated. For a suitable characterization, SEM micrographs of the green, brown, and final parts are presented in Figure 6 at different magnifications. Figure 6a,b exhibits SEM images of the transversal section of an as-printed part. The different printed layers that conform the volumetric geometry can be observed at the lower magnifications (Figure 6a). Between adjacent printed layers, several printing defects, or holes with lack of material, are detected. Two types of voids are observed, the “extrusion voids” that are also presented in the copper and polymer filament, and the “printing voids” that appear between the printed layers. When the filament is extruded through the nozzle, it is strongly sheared and the formation of extrusion voids results from the complex state of stress and from the variation of viscosity as the feedstock spreads out of the extruder and solidifies on the deposited layers. Regarding the printing voids, they are caused and are strongly influenced by the printing strategy followed in the manufacturing of the green parts. The powder particles coated by the binder system, with a similar appearance to that of the filament, can be seen at higher magnifications (Figure 6b). The optimization of parameters such as layer thickness, nozzle speed and extrusion temperature were studied to increase the density values in the green state of copper parts in a previous study [49]. The more optimized results were achieved for a green density of 5.42 g/cm^3^. The suitable parameters used in this work resulted in the manufacturing of green parts with a density of 5.57 ± 0.4 g/cm^3^, which is higher than reported in the previous study. 

After printing, the samples were immersed in solvent to remove the partially soluble wax component. Completion of the solvent debinding stage for the green parts required 10 h. A weight loss of 2.8% has been registered, and the samples had enough strength to be handled. The copper parts after the solvent debinding stage, brown pieces, are shown in Figure 6c,d. The main difference between green and brown stages is that the wax part of the binder system has been removed. Consequently, the agglomeration between the metal powder particles looks less significant and the polymeric film that covered them also becomes less thick. In addition, the limits between the different deposited layers that form the part geometry are less well defined, although pores and holes are still detected. In Figure 6d, it can be seen that adjacent copper particles are still bonded by part of the binder system, probably by the HMW (backbone) polymer, ensuring that these have an enough strength to be handled. Interconnected capillary voids are observed, which can allow the flow of backbone polymer and gaseous decomposition products of degradation of the remained binder. The micrographs of the polished transversal section of a copper part obtained after the complete thermal cycle, which includes the thermal debinding of the HMW binder and the possible remaining soluble wax residue, and the final sintering stage for material consolidation and densification, are shown in Figure 6e,f. No remains of the binder system are observed, so the chemical and thermal debinding are effective and have not negatively interfered in the sintering of the material. The sintering process is driven by an atomic diffusion mechanism, thermally activated. SEM images show a typical metal sintered mesostructure, in which unity and continuous and homogeneous appearance proves the adequate sintering of the copper parts. A low residual microporosity inherent to the sintering process is observed. The micro-pores or interstices are very small and isolated, which denotes that a suitable bonding and a good integrity among copper particles have been achieved after the sintering. 

### 3.2. Mechanical, Thermal, and Electrical Properties

**Figure 7:**
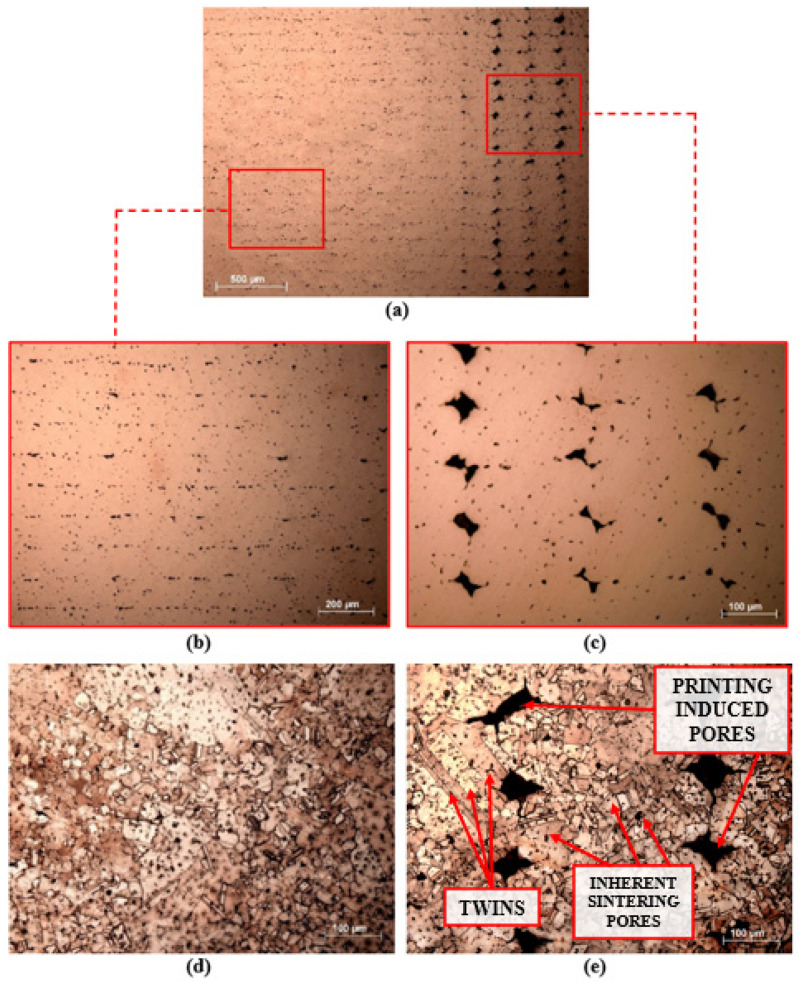
Cross section micrographs of sintered copper parts. (**a**) General view (×50). (**b**) Interior area with solid infill printing strategy (×200). (**c**) External perimeter with contouring printing strategy (×200). (**d**) Microstructure in interior area. (**e**) Microstructure in external perimeter.

Several micrographs of the transversal section of copper sintered parts are presented in Figure 7, which are analyzed based on the microstructure, on the sintering porosity and on the defects provoked by the printing strategy. Different types of porosity can be seen in Figure 7a. The largest pores can be observed on the right side, which corresponds to the external wall of the copper part. These have been induced during the contour printing, based on the followed printing strategy, and could not be completely closed or removed during the sintering process. The detected smaller and more rounded pores are homogeneously distributed in all the transversal section. These are called inherent-sintering pores and usually appear in the metallic parts obtained after a powder metallurgy sintering cycle and are also favored by the inner solid filling printing strategy performed by the print-head. Figure 7b shows the inner aspects of the copper sintered parts, in which it can be seen that the followed solid infill printing strategy is reproduced. This micrograph shows that the porosity distribution is not completely homogeneous or isotropic, with more pores concentrated between layers than within layers. This fact could be expected, as powder particles are not consolidated across layers as well as they are within a layer. Thus, it has been verified that the micropores exhibit a certain alignment at 0.129 mm, adjusting to the layer thickness value established in the printing parameters, and thus having been induced by the printing strategy followed inside the piece. On the other hand, a higher magnification micrograph of the biggest external pores is presented in Figure 7c, in which their irregular geometry and size can be seen. Due to the contouring printing strategy, which follows the layer perimeter during the printing of the established external thickness (4 layers in this study), the outside areas of the copper sintered parts present higher porosity, which notably influences the achieved global density. Moreover, the four layers that form the exterior perimeter sample side can be differentiated thanks to the printing pores induced between them. Despite the printing induced pores remaining relatively large, it can be observed that most of them are rounded, and that they are reduced in size. The fact that bigger cavities divide into smaller pores is an indication that the sintering process is in an advanced stage. 

Figure 7d,e shows the microstructure of the copper sintered parts. Different to the dendritic microstructure of cast copper, copper microstructure obtained by MEAM is polycrystalline based on equiaxed grains. Double parallel straight lines extending across many grains are annealing twins. The details of the copper MEAM parts’ microstructure are indicated in Figure 7e, which was taken on the outside perimeter of the cross section. Besides the printing-induced and the inherent sintering pores, the grain boundaries, as well as the twins within some copper equiaxed grains can be observed.

All the test results, analysis and measurements of the final copper parts obtained in this study are collected in Table 3, as well as Binder Jetting, SLM, EBM and wrought copper values reported in previous research works. It is worth mentioned that mean values and standard deviations are included. For the wrought copper values, the C10100 copper type was chosen as the reference material because it is mainly used in thermal and electrical functional applications. In the case of Binder Jetting, Selective Laser Melting and Electron Beam Melting, the used process parameters and test specifications are defined in the given references. For the wrought material, the values are given with upper and lower thresholds, as this range is due to the section thickness and the temper type applied to the C10100 copper.

As can be seen, similar results to those indicated by Markforged have been obtained in this work. The slight differences can be attributable to the 3D manufacturing process itself. The results obtained in this study are analysed parameter by parameter below. Due to its enormous influence on the properties of the final copper parts, including mechanical, electrical, and thermal properties, the first result to be evaluated is the relative density. The average relative density achieved by the MEAM method, which has been calculated based on the measurements of sintered copper parts with different geometries, is 95.3%. It is noted that the printed geometry does not significantly affect the obtained density measurements, because the registered deviation is about ±0.5%. This fact confirms, regarding density terms, the good reproducibility of MEAM manufactured copper parts.

In 3D manufacturing by high-energy beams, such as EBM and SLM, it is possible to obtain copper pieces with relative densities close to full density, at 99.95 [26] and 99.1% [Ike shoji], respectively. These techniques use additive manufacturing melting technologies in which the powder material is melted to process part, favoring the achievement of almost full density. As it can be seen in Table 3, Material Extrusion Additive Manufacturing is able to produce denser copper parts in comparison with the Binder Jetting process, in which specimens only achieved 85.8% relative density. As both these techniques differ in respect of the printing and sintering steps, their densification stages are based in a thermally activated solid state diffusion process, and there is no melting of powder material, their comparison makes more sense. The higher density results obtained in MEAM technology against BJ means a better powder particle packing in the printing stage that favors the densification in solid-state sintering. Moreover, BJ manufactured parts usually present a limited degree of necking between copper particles, and this provokes the presence of surface-connected and interconnected open pores inside sintered copper products [36]. As can be observed in Figure 7, in the case of copper MEAM parts, both sintering-inherent pores and printing-induced pores are isolated and no communication exists between them, so no open porosity is detected. This fact leads to higher density and predictably better mechanical properties than binder jetting. In addition, the option of using post-processing stages, such as hot isostatic pressing (HIP), can further enhance the final density of copper components manufactured via MEAM technology. Moreover, different microstructures and densities, even higher than those obtained in this work, could be achieved by optimizing both the printing parameters (layer thickness, external wall layers, solid-fill strategy, contouring strategy, etc.) and sintering conditions (temperature, time, protective atmosphere, heating, and cooling rate).

**Figure 8:**
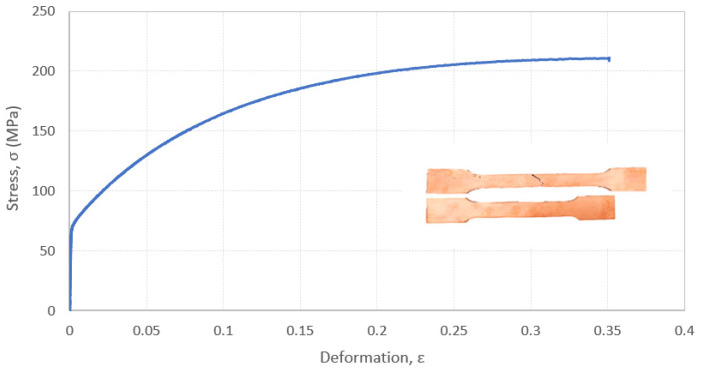
Stress-strain curve of copper Material Extrusion Additive Manufacturing part.

The uniaxial tensile stress-strain curve of an evaluated sintered copper part is shown in Figure 8. The mechanical properties obtained from the different tensile tests were similar and all the samples displayed the same behaviour, which proves a good manufacturing reproducibility. All the tensile curves, including the presented one, include three stages: elastic zone, strain hardening zone and crack propagation zone. In the elastic zone, the stress increases linearly and rapidly until the material started to harden, exhibiting a yielding point at the end of this stage. In the second stage, further stress leads to strain hardening until the ultimate tensile strength is reached. In the third stage the spontaneous and sudden propagation of a crack occurs, after exceeding the highest tensile strength due to the inherent manufacturing process porosity. Note that the stress-strain curve shows in its last stage that copper MEAM samples are very brittle, and their premature fracture is due to the brittleness of the material after hardening, which is induced by the severe propagation of a crack across the pores. 

The achieved values for MEAM manufactured copper parts are near to 65 MPa, 206 MPa and 35% for yield strength, ultimate tensile strength, and maximum elongation percentage, respectively. As can be observed in Table 3, their comparison with other AM techniques and wrought copper demonstrates that the MEAM copper samples processed in this work are suitable and can meet the minimum requirements of wrought parts and high-energy additive manufacturing processes. Furthermore, the long sintering time at high temperature could cause moderated tensile strength and more ductile properties than SLM. In addition, a variety of microstructures and mechanical properties could be achieved by modifying the sintering cycle parameters. Future research could work in this area to adjust the final properties of sintered parts to application requirements.

Comparing with the reported Binder Jetting results, as both techniques are based on the same sintering concepts, it was found that copper MEAM parts attained higher mechanical properties thanks to higher relative density and material integration, which confirms that the strength of sintered copper compacts is proportional to their porosity [Yan]. The highest strength and lowest elongation properties are attributed to SLM parts, due to their higher heating and cooling rates and consequent near to full density parts. Focusing on the SLM and EBM tensile properties, they are attributed to the different cooling rates between these techniques. The high reflectivity of copper to conventional laser light near IR results in low deposition of laser energy in the material that, added to high copper thermal conductivity, provokes high temperature gradients and extremely rapid cooling [Gush]. These phenomena provoke a small grain size microstructure in SLM processed copper specimens that corroborates the higher tensile strength results. In the case of EBM, the used high energy source and its lower reflectivity on copper cause a higher powder bed temperature and slower cooling rates during the manufacturing process, so the obtained microstructure presents bigger grain size than in SLM parts. This translates in the lowest tensile strength together with binder jetting parts, and the highest maximum elongation value [Ike shoji].

The microhardness measurements that have been undertaken for the copper sintered parts and their comparison with the results of wrought copper and other techniques measurements are also presented in Table 3. The microhardness measurements have been taken along the polished cross section of copper samples and avoiding the printing induced porosity, so the Vickers indentations evaluated the copper sintering quality and the influence of sintering inherent microporosity on the material hardness. The microhardness results tend to be similar to the described behavior for the tensile properties. With an average Vickers hardness of 54 HV, MEAM copper specimens present a slightly lower microhardness than those of SLM and wrought copper. Considering that the MEAM technique is an indirect additive manufacturing technique in which the densification is achieved through the sintering process, the obtained hardness values are quite competitive with other copper manufacturing methodologies. Regarding the hardness differences between both SLM and EBM high-energy manufacturing processes, the higher results for SLM copper samples was attributed to the smaller grain size in the microstructure. On the other hand, Vickers hardness was also evaluated on the surface of copper MEAM parts, in order to evaluate the influence of printing voids on the material’s hardness. The presence of printing induced cavities and relatively large-size pores in the contouring layers near the surface (Figure 7) results in a lower average hardness of ~40 HV.

Impact or fracture toughness represents the capability of a material to prevent crack propagation. This property is closely related with ductility: higher ductility results in higher toughness. For materials applied in structural, and even functional, applications, it is optimal to concurrently possess high strength for enduring bigger loads, and high toughness for eluding catastrophic breakdown. The impact toughness can be directly obtained from the absorbed impact energy by Charpy tests and normalized by the cross area. The impact energy absorption is proportional to the closed area of the stress-strain curve presented in Figure 8. As it can be seen in Table 3, the impact toughness for copper MEAM parts is 55 J/cm^2^, which is slightly lower than for wrought copper [Gush]. The lower toughness can be attributed to the presence of defects like porosity (sintering inherent and printing induced), that facilitates the crack propagation and the material failure. However, considering the detected porosity of about a ~5% in copper samples fabricated in this work, the measured impact energy is quite similar to the one assessed for wrought material. This fact corroborates that the main porosity of pure copper MEAM parts is closed and isolated. 

**Figure 9:**
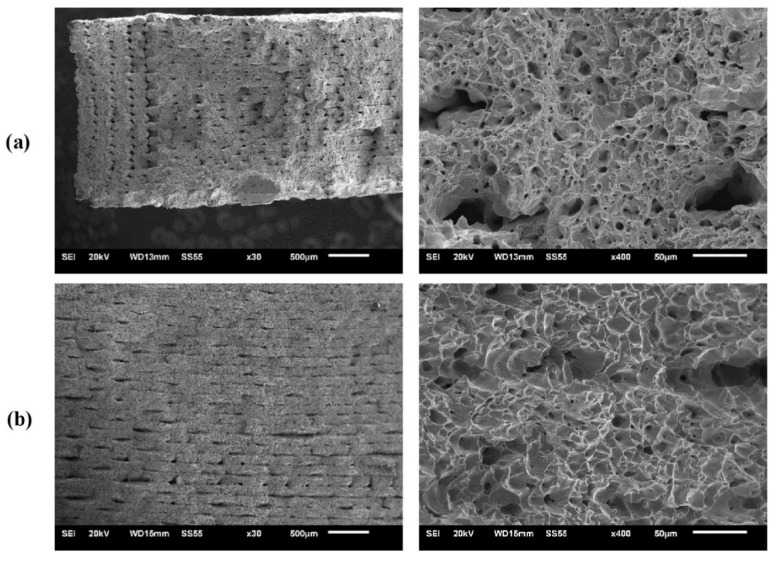
Fracture surface structure of copper MEAM parts. (**a**) Tensile sample. (**b**) Charpy sample.

Micrographs of the fracture surfaces of the copper tensile and Charpy samples obtained by MEAM are presented in Figure 9. Fracture surfaces demonstrate a combination of brittle and ductile fracture, when there are both river patterns and dimples. The fractography indicates a trans-granular failure mode, shaping the usual ductile dimples that appear in soft materials like copper, which is a proof of an excellent cohesive strength between copper particles and high ductility [88]. Thus, the fracture morphology exhibits “honeycomb” shape, as shown in Figure 9. It is also seen that large voids were formed on the fracture surface. Such voids correspond with the pores that can be seen on the cross-section of the sample (Figure 7), which are rather detrimental to the mechanical properties of the material. For copper MEAM samples, the established parameters and printing strategy characteristics result in a poor sintering condition between adjacent layers, which lead to non-closed pores existing after sintering in the interface of the neighboring tracks. Such defects are preferential areas for the generation of stress concentration and initiation of crack, which are propagated causing the fracture of the samples [89]. During the tensile and Charpy tests, the fracture phenomenon easily happened at this layer interface, especially where the pores and voids exist, and its coalescence is inevitable.

Finally, electrical and thermal properties of the copper MEAM parts are presented in Table 3. The Van Der Pauw measurements yielded an average electrical conductivity up to 48·10^6^ S/m, which is equivalent to 82% IACS (International Annealed Copper Standard—definition of 58 µS/m as 100% IACS for electrical conductivity). Laser flash testing yielded a thermal conductivity average of 363 W/mK, which is a relative thermal conductivity of 90%. As can be seen, regarding electrical and thermal conductivities, high values are achieved by MEAM additive manufacturing method. 

Furthermore, both conductivity properties have been evaluated following different directions in the sample, and are nearly identical, so it can be concluded that these physical properties are isotropic for the manufactured pure copper parts. As oxidation and impurities have not been detected, the main objective of this evaluation is to study the effect of the porosity on the thermal and electrical conductivities. In previous research, Vincent et al. [90] studied the effect of the porosity volume fraction on the thermal conductivity of copper parts manufactured via powder metallurgy. A nonlinear evolution of conductivity properties as a function of volume fraction pores was demonstrated, as well as three main domains: Domain-I between 0 and 6% of porosity where a linear decrease of thermal conductivity was observed; Domain-II (transition) between 6 and 9% of porosity where a steep decrease in thermal conductivity was detected; and Domain-III of over 9% of porosity where a linear decrease of thermal conductivity with larger slope than in Domain I was assessed. It is well established that for sintered products, the transition from open to closed porosities occurs for 6% of porosity [91]. Taking into account that the average measured relative density of pure copper parts manufactured in this work was 95.3%, the ~5% of porosity allows these copper specimens to have high thermal and electrical conductivities encompassed by the previously defined Domain-I. As interconnected porosity offers resistance to heat flow (open space), it is verified that the initial open porosity after the debinding stage becomes a reduced isolated (closed) porosity as the densification proceeds in the sintering process. Moreover, the microstructural observations are in agreement with these explanations. 

As the electrical and thermal conductivities are transport properties, it is possible to correct both by considering the sample’s density. There are different expressions to per-form these corrections; however, for high density values,, different corrections will offer similar results [92]. The expression connecting the electrical conductivity with the porosity of a closed-cell material is presented in Equation (4):

(4) $$\;\sigma =\left(1-1.5\cdot \theta \right)\cdot \sigma_{i}$$

where *σ* and *σ_i_* are the electrical conductivity of the sample and the intrinsic conductivity, respectively, and *θ* is the mean samples’ porosity defined by Equation (5), being $\rho /\;\rho_{100}$ the relative density.

(5) $$\theta =1-\rho /\;\rho_{100}\;$$

On the other hand, the thermal conductivity can be related with the porosity through the Equation (6):

(6) $$\lambda =\frac{\left(1-\theta \right)}{1+11\theta^{2}}\cdot \lambda_{i}\;$$

where *λ* and *λ_i_* stand for the thermal conductivity of the sample and the intrinsic one, respectively [93]. 

After considering both corrections we obtain *λ_i_* = 390 W/mK and *σ_i_* = 51·10^6^ S/m for the thermal and electrical conductivities, respectively, which agrees with the IACS tabulated values. Therefore, an improvement in relative density would directly improve both conductivity values. 

Other factors that affect the conductive properties of the solid pure metals are working temperature, atomic lattice structure, impurities, microstructural defects, and anisotropy. In comparison with other AM technologies for copper parts processing, MEAM technique seems to create better conductive properties than SLM and BJ methodologies. The EBM process achieved the best electrical and thermal behavior thanks to having the highest densification and good processability. The notably lower relative density of BJ copper parts compared to MEAM specimens explains the better conductive characteristics for the products manufactured in this study. As reported [Davis], many columnar dendrites with a certain growth direction and an important microstructural anisotropy were detected in SLM copper parts, so thermal and electrical conductivity values are lower than those obtained by MEAM. Thus, microstructural porosity defects that exist in copper parts obtained by Material Extrusion Additive Manufacturing are not the main factors affecting the electrical and thermal conductivities.

## 4. Conclusions

This work studied the geometric, mechanical and functional performance of 3D printed pure copper metal parts manufactured by the cost-effective Material Extrusion Additive Manufacturing (MEAM) technique. The main conclusions are given below:

- Green copper samples manufactured using extrusion 3D printing resulted in a density of 5.57 g/cm^3^, which corresponds with a ~60% of relative density. By an effective solvent debinding stage, a maximum weight loss of 2.8% was observed, which resulted in brown parts with a density of ~5.41 g/cm^3^ and enough strength to be handled. The resulting interconnected porosity provided transport channels for the thermal debinding of the binder system rest, including backbone polymer.
- Pure copper parts with ~95.3% relative density and a ~13.5% approximately isotropic shrinkage were prepared by 3D extrusion printing and sintering with optimized parameters for every process step. This densification result is better than the ones reported in the literature for the fabrication of copper components by indirect additive manufacturing, such as ~83.9% by rapid tooling or 85 to 90% by binder jetting. Moreover, the relative density achieved in the present study is close to those obtained with high-energy beam technologies such as SLM and EBM, which are more complex, expensive and currently developed techniques.
- Tensile strength values of 205.8 MPa and a maximum elongation of 35% by tensile tests, and an average hardness of ~55 HV, were registered for 3D printed copper samples. The good combination of strength and strain led to a high toughness of 55 J/cm^2^, closed to the values reported for wrought copper.
- The achieved mechanical properties are comparable to those obtained by SLM and EBM high-energy technologies, and higher than those for Binder Jetting indirect additive manufacturing methodology. In addition, given that a sintering process is carried out in the manufacturing process, the MEAM technique can achieve specific microstructures by modifying the sintering thermal cycle.
- The measured average thermal conductivity for copper parts fabricated in this work was 363 W/mK, which means a ~90% IACS. The Van Der Pauw test carried out on material extrusion additive manufactured pure copper specimens revealed an average electrical conductivity of 48 × 106 S/m, that corresponds to a ~82%IACS. The MEAM technique attained better thermal and electrical conductive properties than SLM high-energy beam technology and Binder Jetting indirect additive manufacturing methodology. The experimentally achieved thermal and electrical conductivity values are slightly lower than those obtained in EBM and wrought copper, which are ~100% IACS thanks to their higher densification and good processability. The reduction in the measured properties in this study was attributed to thermal and electrical resistance possibly introduced through defects created during the manufacturing process, mainly the printing-induced and the sintering-inherent porosity. As was demonstrated, the porosity effect is more important in the final electrical properties than in the thermal ones.

In short, the use of Material Extrusion Additive Manufacturing technique with subsequent debinding and sintering steps was a cost-effective and promising technology for the manufacturing of copper metal parts with acceptable mechanical, thermal and electrical performance. The overall optimized parameters aim to achieve high density, and they open the way to the manufacturing of pure copper customized parts with high density which are difficult to fabricate with other 3D printing techniques. Furthermore, the MEAM technique can be oriented to the manufacturing of functional copper components that do not usually need fully dense material or are not subjected to extreme loading conditions. In this way, the present technology should allow the fabrication of pure copper customized parts such as complex shaped electric components, optimized-shaped heat sinks for a better heat dissipation in electronic and other devices, or parts with internal cooling channels that are usual in bearing.

## Figures and Tables

**Table 1:** Printing parameters for the additive manufacturing of copper parts by MEAM.

Parameter	Value
Printing Scale	1
Filling Type	Solid infill
Sintered Layer Thickness (mm)	0.129
Exterior Wall Layers	4

**Table 2:** Average values and deviations of the printed and sintered samples dimensions.

Direction	Di CAD Model (mm)	Di Green (mm)	Di Sintered (mm)	% Shrinkage	∆D (%)
*X* axis	55	63.572 ± 0.009	55.151 ± 0.060	13.2	−0.151 ± 0.055
*Y* axis	10	11.462 ± 0.006	9.920 ± 0.012	13.4	0.024 ± 0.003
*Z* axis	10	11.539 ± 0.012	9.988 ± 0.018	13.8	0.080 ± 0.016

**Table 3:** Average values of mechanical, thermal, and electrical properties of copper parts manufactured by MEAM and comparison with other technology values.

Properties	Indirect Processes			Direct/Melting Processes		Wrought [81,82]
	MEAM		Binder Jetting [37,83]	SLM [26,84,85]	EBM [86,87]	
	This Study	Markforged [69]				
Relative Density (%)	95.3 ± 0.5	96–98	85.8 ± 0.4	99.1 ± 0.5	99.95	100
Yield Strength (MPa)	65.0 ± 1.5	26	-	187 ± 5.3	78.1 ± 0.9	69–365
Tensile Strength (MPa)	205.8 ± 5.0	193	176.4 ± 6.5	248 ± 8.5	177 ± 3.3	220–455
Maximum elongation (%)	35.1 ± 1.4	45	28.9 ± 1.6	9.2 ± 1.75	59.3 ± 7.5	4–55
Impact Energy (J/cm^2^)	55 ± 2	-	-	-	-	66.8 ± 1.6
Vickers Hardness (HV)	54.8 ± 2.1	-	-	85 ± 4.2	57.8 ± 1.55	40–130
Thermal Conductivity (W/m·K)	363 ± 9 (90% IACS)	350	245.7 ± 4.7 (61% IACS)	336 ± 7 (84% IACS)	390 ± 5 (100% IACS)	390 (100% IACS)
Electrical Conductivity (×10^6^ S/m)	48 ± 1 (82% IACS)	84% IACS	37 ± 4 (65% IACS)	51 ± 2 (88% IACS)	56 ± 1 (97% IACS)	58 (100% IACS)
